# New Insight into Microbial Exploitation to Produce Bioactive Molecules from Agrifood and By-Products’ Fermentation

**DOI:** 10.3390/foods14081439

**Published:** 2025-04-21

**Authors:** Paola Foti, Cinzia Caggia, Flora Valeria Romeo

**Affiliations:** 1 Consiglio per la Ricerca in Agricoltura e l’Analisi dell’Economia Agraria (CREA), Centro di Ricerca Olivicoltura, Frutticoltura e Agrumicoltura, Corso Savoia 190, 95024 Acireale, Italy; floravaleria.romeo@crea.gov.it; 2 Dipartimento di Agricoltura, Alimentazione e Ambiente (Di3A), Università degli Studi di Catania, Via Santa Sofia, 100, 95124 Catania, Italy; ccaggia@unict.it

**Keywords:** microorganisms, fermentation, bioactive compounds, health benefit, food, by-products

## Abstract

Consumers are increasingly interested in a healthy lifestyle, and choosing foods and ingredients with proven human health benefits has become a current trend. Recently, scientific evidence has proven that the use of microorganisms in different food matrices appears to play a key role in the production of bioactive molecules with biological effects on human health. In particular, selected microorganisms with specific traits can be exploited for the production of specific molecules with high nutraceutical value that can be used in the food industry. This review aims to explore the most recent studies that correlate the use of microorganisms to produce high-value molecules through fermentation and synthetic biology, confirming their strategic role in obtaining nutraceuticals for human consumption with health-promoting effects.

## 1. Introduction

Consumer attention and awareness toward an appropriate and healthy diet focuses on the choice of naturally based, minimally processed foods, such as fermented foods [1]. In the past, fermentation has been considered an effective method mainly for food preservation, while in recent years, the fermentation process has been related to positive changes in the rheological and functional properties of matrices coming from different sources [2]. Therefore, the production of fermented foods is constantly increasing and requires the implementation of different products for the market [3]. Fermented products are commonly defined as ‘foods or beverages obtained through spontaneous or driven microbial growth and subsequent enzymatic transformation of major and minor food components” [4]. Consequently, the selection of suitable microorganisms to produce fermented food and/or ingredients has become a crucial point for production processes at the industrial scale [5]. The use of selected microbial starters allows the fermentation process, avoiding undesirable microbial growth and improving the safety, nutraceutical, and sensory profiles of final products [6]. Overall, microorganisms that best perform as starters are commonly isolated and selected from the native microbiota present in the matrix, as they are already adapted to matrix conditions, to the same interfering factors [7], and even to their own pathogenic microbiota [8]. Several microorganisms and primary metabolites are involved in fermentation processes, such as alcohol and carbon dioxide for yeasts, acetic and lactic acids for acetic acid bacteria and lactic acid bacteria (LAB), propionic acid for *Propionibacterium freudenreichii*, and ammonia and fatty acids for *Bacillus* spp. and molds [4]. It has been recognized that the beneficial effect on human health of fermented foods is related to some molecules produced during the fermentation process, such as enzymes, vitamins, minerals, secondary metabolites, and the microorganisms themselves, which provide additional properties to the basic nutrition of the matrix [9,10]. Moreover, fermenting microorganisms can also turn complex compounds into biologically active metabolites, such as complex polyphenols and flavonoids that are enzymatically hydrolyzed into biologically active molecules [11]. The microorganisms most used as starters are LAB and yeasts for their technological performance and beneficial effects. The healthy effects of LAB and yeasts, in particular *Saccharomyces cerevisiae*, are widely recognized, and a growing interest in their antioxidant activity has been recently registered [12]. In detail, LAB are known to reduce oxidative stress by inhibiting nitric oxide production through a decrease in gene expression of inducible nitric oxide synthase to cyclooxygenase type 2 [13] and for their antineoplastic activity by inducing apoptosis and reducing cancer cell proliferation [14]. In addition, the fermentation process can induce the removal of toxic or harmful compounds such as phytic acid, mycotoxins, and lactose, improving food properties [15]. To improve the efficiency and sustainability of biotechnological fermentation processes, microorganisms can be used individually or in co-culture. Co-culture, which involves the use of two or more microbial species, offers several advantages over single-strain systems. In particular, this strategy can lead to an increase in production yield due to metabolic interactions between the microorganisms, better regulation of product characteristics such as taste, texture, and nutritional content, and the possibility of using less expensive or more complex raw materials [16].

**Figure 1:**
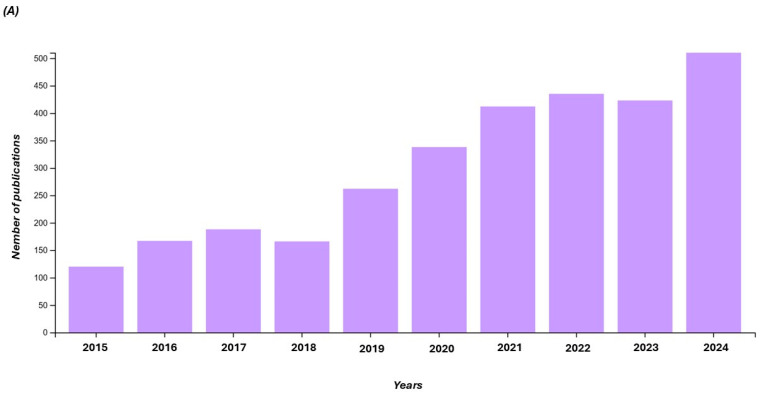

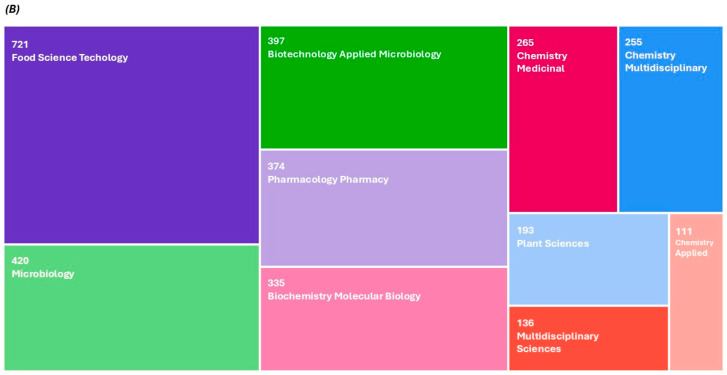
(**A**) Records found on the Web of Science on starter cultures and bioactive compounds during the last 10 years. (**B**) Web of Science categories related to starter cultures and bioactive compounds.

Many researchers have turned their attention to the exploitation of different microbial cultures able to increase the bioactive content of final products. According to recent research performed on the Web of Science, choosing the last ten years (2015–2024) and by typing the words ‘starter cultures’ and ‘bioactive compounds’, 3557 results were found (Figure 1).

In particular, the greatest focus was found on “Food Science Technology” and on “Biotechnology Applied Microbiology”, which shared 70.40% and 15.13% records, respectively, of the total reported from Web of Science categories. The interest in the food sector is mainly related to the improvement of nutritional and rheological properties of food.

**Figure 2:**
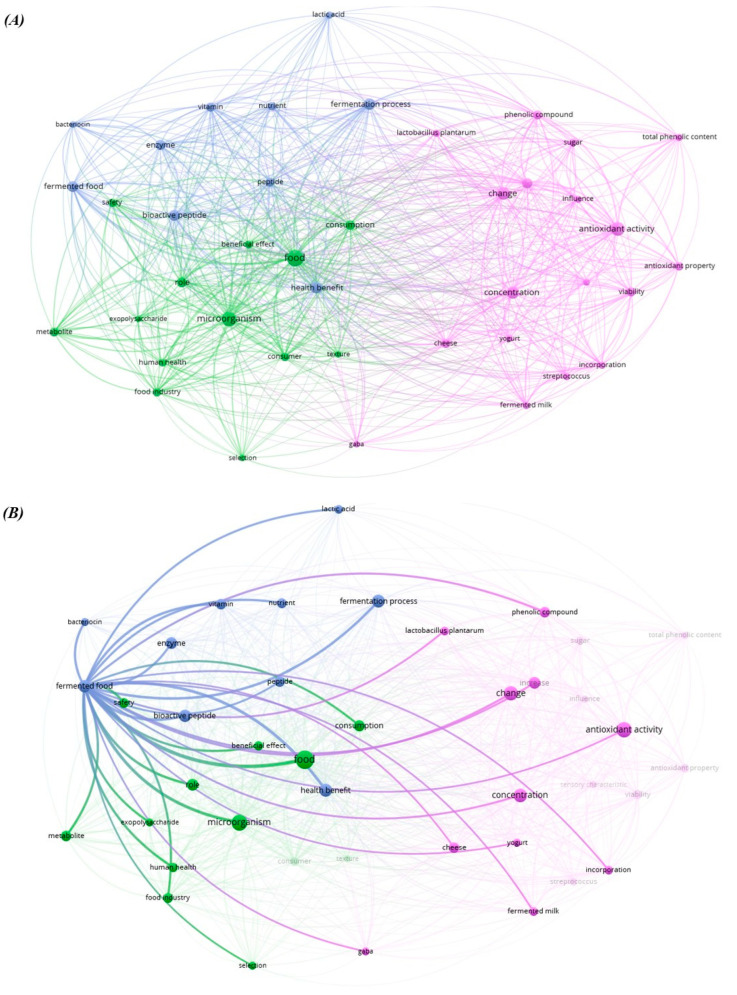
(**A**) Vosviewer network visualization of the bibliometric keywords map for microorganism and bioactive compound papers in food studies from 2015 to 2024 from Web of Science (created in vosviewer.com). (**B**) Vosviewer network correlation with the word fermented food.

A comprehensive survey was conducted on the Web of Science website to highlight the correlation between the words ‘microorganisms’ and ‘bioactive compounds’ (Figure 2). The keyword ‘fermented food’ appears to be linked to the use of LAB and their ability to drive food fermentation, increasing the safety and nutraceutical traits of final products. In detail, during fermentation, compounds with nutraceutical value, such as free phenolic acids, increase as an effect of fiber-bound phenolic acid release, and antioxidant activity can be enhanced by the increase in phenols because of microbial hydrolase activity (Figure 2B) [17,18].

The high demand for the cost-effective production of bioactive compounds at the industrial level requires the involvement of new technologies with low environmental impact. Biotechnological production of bioactive compounds has been intensively studied since the development of cell culture technology, metabolic engineering, systems, and synthetic biology [19]. This last method generally uses genetically modified organisms or tissues instead of wild-type organisms to produce specific compounds in fermenters under controlled conditions. Among the different production systems, the use of selected microorganisms represents a valid strategy to obtain substances with high nutraceutical value. In addition, the application of microbial genome editing techniques, as the clustered regularly interspaced short palindromic repeats (CRISPR)-associated (Cas) system, represents a very interesting tool for agro-food industries to produce antimicrobial substances and bioactive compounds [20]. The ability to modify microbial cells to increase the production of desirable endogenous or exogenous metabolites is an important change in many biotech areas [21]. In the case of genetically modified microorganisms, regulatory agencies in the European Union, the United States, and Canada verify their safety and control the produced compounds to assess and assure food safety [22].

The aim of the present review is to provide an overview of the most recent reports focused on the application of microorganisms to enhance bioactive compound content in food or by-products produced with conventional or biotech-based processes.

## 2. Bioactive Compounds from Fermented Products

### 2.1. Food Fermentation

In recent years, several studies have focused attention on driven fermentation in single or co-cultures of several foods, such as dairy products, cereals, vegetables, and other foods and/or beverages. Fermentation processes can produce a wide range of metabolic products, such as biogenic compounds, fatty acids, organic acids, and bioactive peptides, which can improve the nutraceutical profile of foods (Table 1) [23]. Therefore, the health value of a specific food can be improved through a very rigorous strain selection process based on the type of produced substances.

#### 2.1.1. Dairy Products

Increasing interest is directed toward fermented dairy products thanks to their health effects, in particular, their positive effect on intestinal microbiota [24,25]. Among them, kefir, fermented milk, yogurt, and cheese are known to be a source of probiotic microorganisms [26,27,28]. Several LAB strains have been described to produce high levels of lactic acid, mainly responsible for the specific technological and functional properties of products. Moreover, an additional focus in the dairy sector is the selection of microorganisms with probiotic activities [5,29]. This renewed interest has been linked to the ability of such microorganisms to confer specific health benefits such as hypocholesterolemic, antioxidant, hypotensive, and immunological effects [30,31,32]. Indeed, the use of LAB in dairy matrices induces the production of specific substances such as γ-aminobutyric acid (GABA), several vitamins, bioactive peptides, enzymes, bacteriocins, exopolysaccharides, and conjugated linoleic acid (CLA) as the main bioactive compounds [33]. These compounds induce an increased functional effect by stimulating an immunomodulatory cholesterol-lowering effect, along with antiallergic, antioxidant, and hypocholesterolemic properties, increasing the bioavailability of some nutrients, with anti-obesity, appetite control effects, and prevention of cardiovascular disease.

A recent study explored the impact of using indigenous starter cultures in Moroccan Lben fermented milk. The use of indigenous bacteria, *Lactococcus lactis* subsp. *lactis* and one strain of *Leuconostoc mesenteroides* subsp. *mesenteroides*, in specific conditions (about 8 Log_10_ CFU/mL and temperature at 26.7 °C), modified the nutraceutical profile with a particular effect on the angiotensin-converting enzyme (ACE) activity and GABA content [34]. Similar results were reported by other authors in goat’s milk cheese production. The findings showed a significant increase in ACE inhibitory activity, which plays an important role in blood pressure regulation and antioxidant effect. In detail, the combination of a mixed commercial starter culture (*L. lactis* subsp. *lactis*, *Lactococcus lactis* subsp. *cremoris*, and *Lactobacillus bulgaricus*) has been described as producing the highest antioxidant and ACE-inhibitory activity (62.55%) compared to other microbial combinations. This result confirmed that the development of antioxidant activity during cheese ripening is strain-dependent or a combination of strains selected, indicating differences in peptidase specificity of microorganisms [35]. In addition, a recent study showed that *L. lactis* strains isolated from camel milk induce GABA production through enzymatic decarboxylation of glutamic acid. To date, no GABA-producing strain of *L. lactis* has been isolated from milk, confirming a strong correlation among sources, technological performance, genomic characteristics involved in matrix adaptation, and increase in bioactive content [36]. Authors have discussed that during the fermentation of this product, new bioactive ingredients (peptides or lipids) can be generated [37] that are able to prevent and/or treat obesity and its associated pathologies, such as comorbidities [38]. Bioactive peptides have attracted the attention of several researchers, especially the microbial fermentation of dairy products, correlating a strong relationship between chemical structure and beneficial activity [39]. These properties have been confirmed by Tonolo and co-workers [40], who reported that the antioxidant effect on Caco-2 cells is linked to the increased content of nuclear factor erythroid 2 (Nrf2), which induces overexpression of antioxidant enzymes (superoxide dismutase, thioredoxin reductase 1, or glutathione reductase).

Several reviews have focused on the beneficial effects of fermented milk or yogurt as products containing live bacteria with an active role in the development of new foods responding to the demand for health-boosting products [41]. In detail, it has been confirmed that yogurt produced with *Streptococcus thermophilus* and *L. delbrueckii* subsp. *bulgaricus* shows a higher bioactivity compared to chemically produced yogurt for presenting its characteristic profile of peptides and free amino acids [42]. Meng and colleagues [43] and Chen and co-workers [44] showed that the use of specific starter strains in yogurt implied an in vivo microbial shift of the gut microbiota. Results of these studies showed that fermentation of yogurt with *Lactiplantibacillus plantarum* ZFM55 and *Lacticaseibacillus paracasei* ZFM54 significantly affected the technological and nutraceutical profile of the final product as well as the beta diversity of gut microbiota. In particular, probiotics belonging to *Bifidobacterium*, *Blautia*, *Lactobacillus*, *Ruminococcus*, and *Alistipe* genera showed significant increases in the production of short-chain fatty acids, particularly acetic and butyric acid. Recently, such techniques have been applied to explore characteristic metabolites and functional polypeptides in dairy products, such as fermented milk inoculated with *L. plantarum* L3 [45] and fresh cheese inoculated with probiotic *Lacticaseibacillus rhamnosus* B6, *Limosilactobacillus fermentum* B44, and *L. rhamnosus* KF [46]. A distinctive bioactive peptide profile obtained with *Lacticaseibacillus casei* LBC 237 was found to be crucial for beneficial effects for anti-cancer activity [47].

In this scenario, the exploitation of omics techniques, such as metabolomics and peptidomics, has emerged as a powerful approach to comprehensively understand the complex biochemical changes occurring during fermentation. Through the application of such techniques, recent research has shown that microbiomes or ‘fermentomes’ are taxonomically diverse even in fermented foods themselves, as they are enriched in specific gene clusters potentially associated with specific health activity. These advances, combined with an increasing number of in vivo studies, have led to new insights into the mechanisms by which fermented products positively modulate the gut microbiota, the gut/brain axis, and metabolic health [48,49].

#### 2.1.2. Vegetables or Plant-Derived Products

In vegetables or plant-derived foods, which are rich in health-promoting compounds, driven fermentation enhances the conversion of phenolic compounds, such as flavonoids, to biologically active metabolites through the expression of glycosyl hydrolases, esterases, decarboxylases, and phenolic acid reductases. It has long been recognized that phenolic compounds exert a beneficial effect on human health, related to the microbial ability to metabolize such compounds through decarboxylation and/or reduction activities [50] that, in turn, increase their bioavailability [51]. Furthermore, fermentation with specific starters has been shown to influence the content of vitamins, GABA, bioactive peptides, and organosulfur compounds. Among fermented vegetables, cocoa fermentation is one of the most explored processes to obtain a high-quality product. Driven fermentation of cocoa beans results in the formation of aromatic and tasty compounds and contributes to the formation of bioactive molecules with antioxidant, antihypertensive, anti-diabetic, anti-Alzheimer’s, anti-obesogenic, and anti-tumor effects [52]. Authors investigated that *Pichia kudriavzevii* in single culture or in combination with *Saccharomyces cerevisiae* (equal proportions 1:1) induced higher contents of phenolic compounds and methylxanthines in cocoa compared to the control, resulting in a higher antioxidant activity. Furthermore, the synergy between the two starters inhibited the production of putrefactive amines and increased phenylethylamine, a mood-modulating amine [53]. Xu and co-workers [54] showed that plant and animal-derived *Pediococcus pentosaceus* spp. in broccoli juice fermentation positively influenced the composition of bioactive compounds (glucosinolates, sulforaphane, and sulforaphane nitrile), improving the organoleptic properties of the product. Lee and colleagues (2023) [55] evaluated the health effects of a vegetable juice consisting of *Brassica oleracea* var. *capitata*, *Brassica oleracea* var. *italica*, *Daucus carota* L., and *Beta vulgaris* fermented with *Companilactobacillus allii* and *Lactococcus lactis* isolated from kimchi. Results showed that the fermented juice reduced lipid accumulation in human adipose-derived mesenchymal stem cells differentiated from adipocytes through modulation of cytokines and that some metabolites, such as indole-3-lactic acid, leucic acid, and phenyllactic acid, showed an inhibitory effect on in vitro lipid accumulation by significantly decreasing the expression of adipogenesis regulators. In addition, Mantzourani et al. [56] found a strong correlation between the use of two LAB, *Lactiplantibacillus paracasei* SP5 and *P. pentosaceus* SP2, and phenolic content in two juices consisting mainly of red fruits (the first juice contained red apple, orange, pomegranate, and red grape; the second fruit juice included red apple, red grape, cherry, pomegranate, sour cherry, strawberry, black chokeberry, blueberry, blood orange, and black carrot). After fermentation, the content of total phenols, zeaxanthin, lutein, β-carotene, and antioxidant activity was found to be higher than in the controls. In detail, the increased β-carotene content during fermentation was related to the release of carotenoids as an immediate response to macromolecular changes in the juice and the increased synthesis of carotenoids produced in response to oxidative stress. Furthermore, the chemical composition of curly kale fermented with native LAB (to obtain juice) was analyzed by Szutowska et al. [57], showing a close correlation between the increase in a specific substance and the LAB strain used. In detail, a higher content of riboflavin and pyridoxine, compared to the control, was detected in juice fermented with *Latilactobacillus sakei,* while in juice fermented with *L. plantarum*, an increase in pyridoxine and a decrease in riboflavin was observed. Furthermore, it was shown that the fermentation of broccoli carried out with LAB (*Levilactobacillus brevis* and *Lactococcus lactis* as initial co-culture) induced a change in glucosinolate composition and phenolic content. The extract obtained from fermented broccoli exhibited increased antioxidant activity in Caco-2 cells and inhibited proliferation of HT29 and HT116 cell lines in a concentration-dependent manner [58]. Septembre–Malaterre et al. [17] suggested that the conversion of these compounds is due to the esterase activity of LAB, which catalyze the hydrolysis of ester groups, enhancing the bioavailability of phenolic acids and flavonoids. Moving to cereals, another suitable matrix for driven fermentations, it has been shown that different grain substrates, fermentation conditions, time, and used microorganisms can affect the content of different metabolites [59]. In a recent study, wheat bran was fermented with *Eurotium cristatum*, one of the most abundant fungi in fermented tea. The results showed an increase in dietary fiber content and bioactive molecules, such as polyphenols, anthocyanins, and the main aromatic substances. In particular, phenylethyl alcohol increased significantly, with *E. cristatum* at a density of 5 × 10^8^ spores/mL compared to the unfermented sample [60]. Furthermore, fermentation was shown to produce β-hydroxy acid metabolites of monacolin K, an anti-inflammatory, antioxidant, neuroprotective, and anti-tumor compound, able to induce apoptosis and reduce, in a dose-dependent manner, the growth of gastric cancer cells by inhibiting the expression of histone deacetylase enzyme [61]. Furthermore, bacterial strains isolated from fermented soya, belonging to *Bacillus* spp. and *L. delbrueckii* subsp. *bulgaricus*, have been used for the fermentation of soya combined with a commercial strain of *Wickerhamomyces anomalous* [62]. An increase in peptide, total phenol and total flavonoid content, antioxidant activity, and organic acids were detected. Furthermore, the in vivo test highlighted that the fermentation induced high hepatic glycogen levels, improved antioxidant enzyme activity, and decreased malondialdehyde (MDA) levels in serum and liver. Finally, Sidari et al. [63] used strains of *Lactobacillus sanfranciscensis* B450, *Leuconostoc citreum* B435, and *Candida milleri* L999 in sourdough, obtaining an increase in phenolic and antioxidant content.

**Table 1:** Effect of fermentation on food biological activity.

Sector	Matrix	Starter Cultures	Main Effects	References
Dairy products 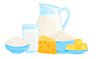	Lben	*L. lactis* subsp. *lactis* and *L. mesenteroides* subsp. *mesenteroides*	Increased angiotensin-converting enzyme (ACE), Inhibitory activity γ-aminobutyric acid (GABA)	[34]
	Goat’s milk cheese	*L. lactis* subsp. *lactis*, *L. lactis* subsp. *cremoris* and *L. bulgaricus*	Increased angiotensin-converting enzyme (ACE) inhibitory activity	[35]
	Yogurt	*S. thermophilus* and *L. delbrueckii* subsp. *bulgaricus*	Increased peptides production	[42]
	Camel milk	*L. plantarum* ZFM55 and *L. paracasei* ZFM54 and *L. lactis*	Increased amino acids and carbohydrate content Increased the metabolite content Increased bioactive content	[36]
	Milk	*L. plantarum* L3	Improved taste and nutritional qualities of milk	[47]
	Fresh cheese	*L. fermentum* B44 (CGMCC 17321) *L. rhamnosus* KF7 (CGMCC 6430) *L. rhamnosus* B6 (CGMCC 13310)	Increased peptide production	[48]
Vegetables, fruits and derivatives 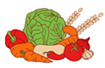	Cocoa bean	*Pichia kudriavzevii* in single culture or in combination with *S. cerevisiae*	Increased phenolic compounds	[53]
	Broccoli juice	*Pediococcus pentosaceus*	Increased composition of bioactive compounds	[54]
	Vegetable juice	*Companilactobacillus allii* and *L. lactis*	In vitro inhibition of lipid accumulation	[55]
	Vegetable juice	*L. paracasei* SP5 and *P* *ediococcus pentosaceus* SP2	Increased phenolic compounds, total phenols, zeaxanthin, lutein, β-carotene content	[56]
	Curly kale	*L. sakei* and *L. plantarum*	Increased content of riboflavin and pyridoxine and pyridoxine and decreased riboflavin content	[57]
	Fermented broccoli	*L. brevis* and *L. lactis*	Increased phenol content and antioxidant activity in Caco2	[58]
	Wheat bran	*Eurotium cristatum*	Increased dietary fiber content and bioactive molecules	[60]
	Fermented soya	*Bacillus* spp. *L. delbrueckii* subsp. *bulgaricus* and *Wickerhamomyces anomalous*	Increased peptide production, total phenol and total flavonoid content, antioxidant activity, organic acids and biological amines	[62]
	Sourdough	*L. sanfranciscensis*, *Leuconostoc citreum* and *Candida milleri*	Increased phenolic and antioxidant content	[63]

### 2.2. Agrifood By-Product Fermentation

**Figure 3:**
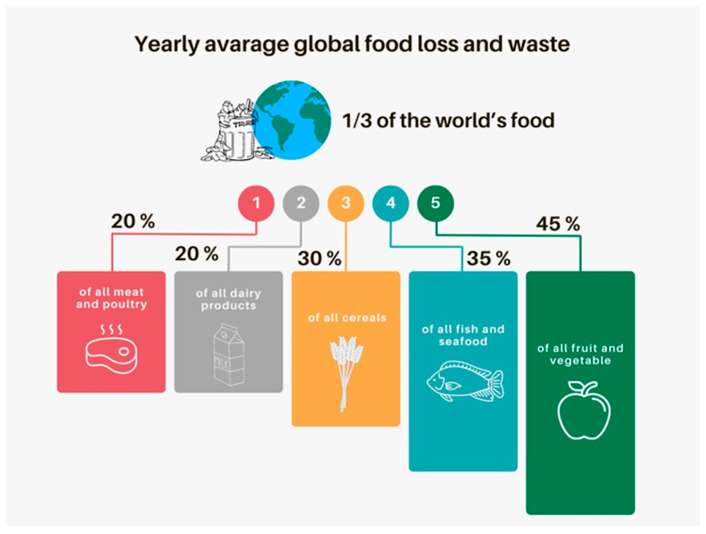
Graphic realized with slight modifications from www.ifco.com/countries-with-the-least-and-most-food-waste/ (accessed on 15 February 2025).

Agrifood by-products are substances originating during various phases of agricultural operations and food processing that still retain several bioactive components, which can be used to develop innovative food products [64]. Several techniques have been proposed to valorize these matrices; one of the most interesting is fermentation through selected microbial cultures [65]. Such a strategy is the key to the implementation of a sustainable bioeconomy inspired by the valorization of by-products resulting from the agrifood industry, which is highly related to the production activities of a territory [66]. Although several chemical-based extraction methods have been proposed for the recovery of bioactive molecules from agrifood by-products, each with different advantages concerning speed and efficiency, none of them can provide the specific benefits associated with microbial cultures. As a matter of fact, the valorization of by-products through the fermentation approach represents a sustainable and eco-friendly solution. As reported by Sabater et al. [67], by-product fermentations are mainly driven by bacteria or fungi that promote the production of bioactive compounds or the improvement of nutritional and sensory traits of the obtained products that could be used as new functional foods or ingredients. By means of driven fermentation, bioactive compounds are obtained as microbial secondary metabolites [68], and the bioactive content is modified through the fermentation process. In general, LAB such as *Streptococcus thermophilus*, *Lactococcus lactis*, *Leuconostoc* spp., *Pediococcus* spp., *Oenococcus* spp., and *Weissella* spp. and fungi such as *Aspergillus* spp. are used in most cases [69]. According to the Food and Agriculture Organisation (FAO), the largest amounts of by-products are generated from fruit and vegetable processing waste (45%), followed by fish and seafood (35%), cereals (30%), dairy products (20%), and meat (20%) (Figure 3).

Therefore, the valorization of by-products from fruits and vegetables is of considerable interest (Table 2). In detail, several authors have proposed the fermentation of by-products such as orange peels, molasses, argan press cake, and carob pods through the use of specific LAB, such as *L. casei* 2264, *L. paracasei* NRRL B-4564, *L*. *plantarum* Argan-L1, and *L. rhamnosus* ATCC 53103, respectively, for the production of lactic acid [70,71,72,73]. Therefore, the lactic acid obtained can be used as an acidifier in the food/beverage sector to produce soft drinks, candies, baked goods, dairy products, jams, and jellies. Furthermore, Sarris et al. [74] showed how the fermentation of olive mill wastewater with *Yarrowia lipolytica* can produce other organic acids, such as citric and oleic acid. Indeed, Romeo et al. [75] and Lazzaroli et al. [76] demonstrated that the application of specific strains of LAB and yeasts, such as *L. plantarum* and *W. anomalus*, or the symbiotic culture of microorganisms was effective in increasing hydroxytyrosol starting from the phenolic extract obtained from olive mill wastewater and olive leaves. In addition, driven fermentation using microbial pools isolated from table olives (*L. plantarum*, *W. anomalus*, and *C. boidinii*) in fermented olive mill wastewater or patè olive cake has been shown to increase the concentration of hydroxytyrosol and, consequently, the antioxidant effect, revealing a different modulation for anti-inflammation on cyclooxygenases and permeability activity on Caco-2 tumor cell lines. This latter effect appeared dependent on the strain used and on the interaction between microbial strain and phytocomplex present in the plant matrix [77,78]. In addition, Cheng et al. [79] evaluated the fermentation of blueberry pomace with a probiotic *L. casei* and demonstrated a strong correlation between increased antioxidant activity and polyphenol content. Falah et al. [80] stated that both the selected strains with specific traits and the specific substrate are essential for an optimal result, demonstrating how the production of GABA could be improved with specific ratios of by-products (molasses, dairy sludge, and soybean meal) inoculated with three bacteria: *Lactobacillus brevis* PML1, *L. fermentum* 4-17, and *L. plantarum* 1058. They found that the optimal treatment included a culture medium containing 29.27% dairy sludge, 24.77% molasses, and 10.49% soybean meal fermented for 48 h with *L. fermentum* 4-17. Under such conditions, they obtained the highest concentration of GABA (359.45 ppm), demonstrating the highest antimicrobial, antioxidant, and anti-tumor activity. Furthermore, through fermentation by specific microorganisms, authors have shown how fiber, protein, and sugar content can be modified to propose a new booster for food applications. In detail, different species have been applied for the valorization of several by-products, such as *Trichoderma* spp. in pineapple peels and *L. lactis* in combination with *Hanseniaspora apuntiae* in pineapple cores [81,82], *Weissella*, *Saccharomyces*, *Aspergillus oryzae*, and *Zygomycetes fungi* in apple by-products [83,84,85,86], *L. plantarum* T6B10 and *Weissella confusa* BAN8 in maize, *L. plantarum* LB1 and *L. rossiae* LB5 in hemp and chickpea by-products [87,88], *Rhizopus* in rapeseed press cake [89], *Aspergillus oryzae*, *Pleurotus ostreatus*, and *Hericium erinaceus* in lime-cooked maize, and *Fusarium flocciferum* and *Rhizodiscina* cf. *lignyota* in olive cake [90,91].

### 2.3. Engineered Microorganisms for Bio-Production of Active Compounds

Exploitation and engineering of microorganisms to produce bioactive compounds such as nutraceuticals and biopharmaceuticals is of growing interest [92]. The CRISPR-Cas system has rapidly emerged as a powerful tool for genome engineering, offering high efficiency, precision, and flexibility, and has revolutionized traditional approaches in microbial biotechnology [93]. Indeed, this method is useful for overcoming issues associated with the conventional extraction of bioactive compounds from matrices and increasing the yield of specific molecules. Therefore, to increase the heterologous production of highly active plant metabolites, the biosynthetic pathways must be known so that specific microbial cell factories can be developed, providing a source for large-scale production of plant-derived bioactive compounds [94]. Among the microorganisms to be genetically engineered for producing natural compounds de novo or semi-de novo, *S. cerevisiae* and *Escherichia coli* [95] have been mostly used.

To date, *S. cerevisiae*, considered Generally Recognised As Safe (GRAS), is the most widely used microorganism to produce bioactive molecules. In detail, its widespread use is related to its flexible genome, its protein content, and the formation of antioxidant compounds, including glutathione [96,97]. Guo et al. [98] demonstrated how, through the engineering of specifically related enzymes, the production of triterpenoids was significantly improved from milligram per liter to gram per liter. These results confirmed that the engineered enzyme increased the 2,3-oxydosqualene precursor, optimized the reactions involving triterpenoids, and reduced the competition of the native sterol pathway. To optimize the timing of strain selection and production, Gowers and co-workers [99] proposed the SCRaMbLE system as an in vivo deletion and rearrangement system of synthetic yeast chromosomes that can produce strains with improved biosynthetic phenotypes to produce triterpenoids, especially betulinic acid [99]. Furthermore, by engineering *S. cerevisiae*, the authors increased the yield of ginsenoside, which is recognized as a major component of ginseng and a potential candidate for arthritis therapy. The authors showed that by improving the biosynthesis of uridine diphosphate glucose and reducing its consumption, the engineered yeast produced 5.74 g/L of ginsenoside [100]. Chen et al. [101] showed that through the modulation of three of the most common cofactors (FADH2, S-adenosyl-l-methion, and NADPH), the production of phenolic acids in *S. cerevisiae* was highly improved, specifically the production of caffeic acid and ferulic acid, precursors of many pharmaceutical molecules, with a concentration of 5.5 ± 0.2 g/L and 3.8 ± 0.3 g/L, respectively. Regarding *E. coli*, the greatest advantages are related to the rapid growth and the ease of genetic manipulation. Several research groups have extended the native pathway of *E. coli* by adding heterologous genes to produce several metabolites. Different compounds are generated through metabolic manipulation, such as polyunsaturated fatty acids, polyphenolic compounds, carotenoids, and non-proteinogenic amino acids [102]. In recent years, several studies have shown how specific strains of *E. coli*, in single or in co-culture, under certain growth conditions and with specific carbon sources, can produce specific compounds, such as docosahexaenoic acid [103], naringenin [104], resveratrol [105], astaxanthin [106], and GABA [107].

Although CRISPR-Cas and synthetic biology offer powerful tools for microbial engineering and new functional food development, their implementation is subjected to restrictions [93]. Potential off-target effects, stability of engineered traits, and unintended ecological consequences are still areas of active investigation. Moreover, biosafety concerns must be rigorously addressed through standardized risk assessments and containment strategies, particularly when genetically modified organisms are introduced into food systems [108]. Furthermore, ethical considerations, such as the transparency of genetic modifications, equitable access to technology, and the right of consumers to make informed choices, underscore the need for a robust, multidisciplinary framework. As such, future progress in this field must balance innovation with responsibility, ensuring that technological advancements are aligned with societal values and safety standards [109].

## 3. Conclusions and Future Perspective

The food industry aims to implement new technologies for the development of specific microbial cultures to improve the nutraceutical profile of foods or agrifood by-products. In this context, the exploration of microorganism/matrix interaction through fermentation or engineering techniques represents a key approach to obtaining bioactive molecules from different matrices. Furthermore, through metabolomic approaches and metabolic engineering of specific microorganisms, it is possible to develop new functional foods or food supplements with specific biological characteristics with a targeted health-promoting effect. These approaches, therefore, represent an environmentally friendly, sustainable solution for the industrial production of novel foods and nutraceuticals with high health value. Regarding these kinds of productions, especially for by-products, before placing a product on the market, safety, quality, and clear labeling of the new food or supplement must comply with food legislation. In addition, regarding synthetic biology, future developments in this field will need to deal with several challenges, such as the evolution of regulatory frameworks that keep in step with scientific innovation and the promotion of social acceptance of engineered microbes in food systems.

## Figures and Tables

**Table 2:** By-products and waste valorization through fermentative processes.

Agro-Food By-Products	Starter Cultures	Main Results	References
Orange peels	*L. casei* 2246 (single culture) *L. plantarum* 285 and *L. paracasei* 4186 (mixed cultures)	Lactic acid production in solid state fermentation	[70]
Molasses	*L. paracasei* NRRL B-4564	Lactic acid production in solid state fermentation	[71]
Argan press cake	*L. plantarum* Argan-L1	Increased antioxidant activity related to lactic acid and protein content	[72]
Carob pods	*L. rhamnosus* ATCC 53103	Improved lactic acid production and yield	[73]
Olive mill wastewater	*Yarrowia lipolytica*	Production of citric and oleic acid	[74]
	*L. plantarum* and *W. anomalus*	Increased hydroxyditorosol content	[75]
	*L. plantarum*, *W. anomalus* and *C. boidinii*	Increased hydroxyditorosol content and biological activity	[77]
Olive leaves	*Commercial SCOBY*	Increased hydroxyditorosol and oleuropein content	[76]
Blueberry pomace	*L. casei*	Increased antioxidant activity and polyphenol content	[79]
Molasses, dairy sludge, and soybean meal	*L. brevis* PML1, *L. fermentum* a *L. plantarum* 1058	Production of valuable medicinal and bioactive compounds	[80]
Pineapple peels	*Trichoderma*	High protein production	[81]
Pineapple core	*Lactococcus lactis LA5*, *Hanseniaspora* *opuntiaeSA2*	Production of new fermented food with high fiber and amino acid content	[82]
Apple by-products	*Weissella cibaria* PEP23F *Saccharomyces cerevisiae* AN6Y19	Production of a functional ingredient for wheat bread	[83]
	*Aspergillus oryzae*	Production of a functional ingredient with antioxidant and prebiotic activity	[84]
	*Saccharomyces cerevisiae*	Production of new fermented beverages with antioxidant activity	[85]
	*Zygomycetes fungi*	Production of a functional ingredient with a high level of phenol	[86]
Maize milling by-products	*L. plantarum* T6B10 and *Weissella confusa* BAN8	Production of a functional ingredient for bread	[87]
Hemp, chickpea, and milling by-products	*L. plantarum* LB1 and *L. rossiae* LB5	Production of a functional ingredient for pasta	[88]
Rapeseed press cake	*Rhizopus microsporus* var. *oligosporus*	Production of a functional ingredient	[89]
Lime-cooked maize	*Aspergillus oryzae*, *Pleurotus ostreatus*, and *Hericium erinaceus*	Increased free phenolic compounds, antioxidant capacity, and soluble dietary fiber	[90]
Olive cake	*Fusarium flocciferum*, *Rhizodiscina* cf. *lignyota*	Production of efficient enzymes leading to nutritional enhancement of this by-product	[91]

## Data Availability

No new data were created or analyzed in this study. Data sharing is not applicable to this article.
